# Role of miRNAs in regulation of SA-mediated upregulation of genes involved in folate and methionine metabolism in foxtail millet

**DOI:** 10.3389/fpls.2022.1023764

**Published:** 2022-12-06

**Authors:** Siyu Hou, Yihan Men, Yijuan Zhang, Kai Zhao, Guifang Ma, Hongying Li, Yuanhuai Han, Zhaoxia Sun

**Affiliations:** ^1^ College of Agriculture, Institute of Agricultural Bioengineering, Shanxi Agricultural University, Taigu, Shanxi, China; ^2^ Shanxi Key Laboratory of Minor Crops Germplasm Innovation and Molecular Breeding, Shanxi Agricultural University, Taiyuan, Shanxi, China

**Keywords:** foxtail millet, salicylic acid, miRNA, folate, methionine metabolism

## Abstract

The effect of exogenous salicylic acid (SA) on folate metabolism and the related gene regulatory mechanisms is still unclear. In this study, the panicle of foxtail millet treated with different SA concentrations showed that 6 mM SA doubled the 5-methyltetrahydrofolate content compared to that of the control. An untargeted metabolomic analysis revealed that 275 metabolites were enriched in amino acid metabolic pathways. Significantly, the relative content of methionine (Met) after 6 mM SA treatment was 3.14 times higher than the control. Transcriptome analysis revealed that differentially expressed genes were mainly enriched in the folate and amino acid biosynthesis pathways (including Met, Cys, Pro, Ser et al.). The miRNA−mRNA interactions related to the folate and Met metabolic pathways were analyzed and several likely structural gene targets for miRNAs were identified, miRNA-seq analysis revealed that 33 and 51 miRNAs targeted 11 and 15 genes related to the folate and Met pathways, respectively. Eight key genes in the folate metabolism pathway were likely to be up-regulated by 14 new miRNAs and 20 new miRNAs up-regulated the 9 key genes in the Met metabolism pathway. The 6 miRNA−mRNA interactions related to the folate and Met metabolism pathways were verified by qRT−PCR, and consistent with the prediction. The results showed that *DHFR1* gene expression level related to folate synthesis was directly up-regulated by Nov-m0139-3p with 3.8 times, but *DHFR2* was down-regulated by Nov-m0731-5p with 0.62 times. The expression level of *CYSC1* and *APIP* related to Met synthesis were up-regulated by Nov-m0461-5p and Nov-m0664-3p with 4.27 and 1.32 times, respectively. Our results suggested that exogenous SA could induce the folate and Met accumulated in the panicle of foxtail millet. The higher expression level of *DHFR1*, *FTHFD*, *CYSC1* and *APIP* in the folate and Met metabolism pathway and their regulators, including Nov-m0139-3p, Nov-m0717-5p, Nov-m0461-5p and Nov-m0664-3p, could be responsible for these metabolites accumulation. This study lays the theoretical foundation for elucidating the post-transcription regulatory mechanisms of folate and Met metabolism.

## Introduction

Foxtail millet (*Setaria italica* (L.) P. Beauv.), which originates from northern China, has been cultivated and used for 10,000 years according to archaeological evidence (Lu et al., 2009; Yang et al., 2012). At present, its small genome size, short growth cycle, stress tolerance and good nutritional value have made it a C4 research model plant for exploring some essential gene functions (Yang et al., 2020). The diverse foods made of foxtail millet grains, rich in starch, protein and vitamins, are popular in China, India and some developing regions. Folates (VB9) comprise a pteridine ring, a para-aminobenzoic acid (ρ-ABA) and an L-glutamate tail. They are water-soluble B vitamins tetrahydrofolate (THF) and its derivatives (Hanson and Gregory, 2011). As one-carbon (1C) donors, folates play a central role in 1C metabolism in plant development because they participate in a series of methylation reactions and amino acid metabolism (Hanson and Roje, 2001; Strobbevan der Straeten, 2017). Folate deficiency in developing regions is a common phenomenon because staple foods are preferred in the daily diet (Gamble et al., 2005; Gorelova et al., 2017). Compared to wheat, rice and maize, with folate contents ranging from 6 to 43 μg/100 g, the average folate content is greater in the grains of foxtail millet, which contains 200 μg/100 g. Previously, we determined folate derivatives in the panicle of foxtail millet during the ripening stage and found that the major folate compound is 5-methyltetrahydrofolate (5-M-THF). Moreover, the *SiADCL1*, *SiGGH* and *SiFBP* genes were associated with folate content in foxtail millet (Hou et al, 2022a, b). However, the gene regulation mechanism of folate metabolism in foxtail millet is still unclear and needs further exploration.

Salicylic acid (SA) is an endogenous molecular signal in plants that is widely involved in regulating plant growth and development *via* a series of physiological processes. A previous study showed that after 24 h of 0.25 mM SA treatment of coriander leaves, the contents of folate and its derivatives were significantly enhanced by 2~6-fold compared to the control (Puthusseri et al., 2013). Another report revealed that after SA treatment, the folate content improved two-fold compared with the control, and the folate binding protein expression level was enhanced in *Arabidopsis* leaves (Puthusseri et al., 2018). These reports suggest that SA plays a crucial role in regulating folate synthesis in plants. Previously, a few researchers reported that SA treatment improved protein content and increased amino acid components, including methionine, phenylalanine, threonine, glutamic acid, proline and tyrosine (Farhangi-Abriz and Ghassemi-Golezani, 2016; Zanganeh et al., 2018). Meantime, as well known, the SAM (S-adenosylmethionine) as a 1C product in folate 1C metabolism shared a metabolic step with methionine synthesis through methyl group transferring reaction(Sauter et al., 2013). Hence, we hypothesized the SA treatment could affect folate and methionine level in the panicle of foxtail millet. MicroRNAs (miRNAs), are a group of noncoding single-stranded RNAs responsible for regulating target gene expression at the translation level. They have been proven important in sustaining genome integrity, plant growth development and abiotic stress responses (Jin et al., 2013; Baldrich et al., 2018; Sun et al., 2019). In cotton, the overexpression of miR160 improves the content of indole-3-acetic acid, regulates the length of fiber cells and affects cotton fiber development (Liu et al., 2019). Leaf and flower development in tomatoes were promoted by miRNA160a, which regulates SlARF10A as its target gene (Damodharan et al., 2018). In addition, miR396a-5p or miR396a-3p negatively regulated downstream target genes related to SA and MeJA signaling pathways and could improve the resistance to Phytophthora capsici infections in tomatoes (Hong et al., 2019). According to these previous reports, the expression of some key genes involved in the folate synthesis pathway correlated with folate content. However, the mechanism of SA-regulated folate synthesis and Met metabolism in foxtail millet is still unclear. Meantime, miRNAs regulated the expression of folate or amino acid synthesis pathway-related genes under SA induction needs to be explored and studied further. Hence, we tried to elucidate the effects of SA treatment on metabolites in the panicle of foxtail millet, exploring differentially expressed genes related to the folate and Met metabolism pathways. We predicted the key miRNAs regulating these genes related to the folate and Met metabolism pathways and constructed a mechanistic model of the mechanism of miRNA−mRNA interaction network of the role of SA regulation of the folate and Met metabolism pathways in foxtail millet.

## Materials and methods

### Plant materials and SA treatments

The foxtail millet variety B24 was donated from Shanxi Agricultural University and planted in the experimental field (112°28’E, 37°12’N) from May to October 2020. When the plants grew to the fertilization stage, 0, 3, and 6 mM SA treatments were sprayed onto the panicle of the foxtail millet every 10 days. All SA treatments were performed 3 times during three panicle development stages: S1 (grain-filling stage), S2 (grain middle maturation stage) and S3 (grain maturation stage). Twenty-four hours after the third SA treatment, the panicles were collected and frozen in liquid nitrogen for further folate determination and untargeted metabolome analysis.

### Folate content determination

All samples were ground into a powder using liquid nitrogen with a grinding machine. A total of 0.3 g of powder from each sample was put into a 2 mL centrifuge tube and 1 mL extraction solution (containing 50 mM phosphate buffer solution, 2% ascorbic acid and 10 mM DTT, pH=6.8) was added. After boiling for 10 min, the extraction solution was placed into an ice water bath for another 10 min. Then, 100 μL rat serum was added to the extraction solution and incubated at 37 °C for 2 h. Subsequently, the extracts were placed in a boiling water bath for 10 min, followed by cooling on ice and centrifugation at 13,000 rpm for 30 min. Finally, the treated extracts were filtered through a 3 KDa microporous membrane and were ready for LC-MS analysis. The method of determining the folate derivatives (FA, THF and 5-MTHF) by LC-MS was described by Wan et al. (2019). The standards were ordered from Solarbio Life Sciences Co. (Beijing, China).

### Untargeted metabolome analysis

Two equally-sized powder samples were weighed to 0.1 g and placed into 2 mL centrifuge tubes for metabolite extraction. For water-solubility and lipid-solubility metabolite extraction, 1 mL of 70% and 100% methanol was added to one of the samples, and the samples were incubated at 4°C overnight after vortex blending. Subsequently, 1:1 proportions of the water-soluble and lipid-soluble metabolite extraction solutions were blended at 4°C and centrifuged at 14,000 rpm for 5 min. The lipid supernatant of the extracts was filtered with 0.22 Millipore filter. The metabolites in the extract samples were determined by the Q Exactive TM Plus platform. The metabolites were analyzed by extracting the positive ion mass spectra and using the online tool MetaboAnalyst (https://www.metaboanalyst.ca/) to predict the metabolite species, compound structural formula and molecular weight. The relative content of metabolites, including (Arg, Met, Pro, Ser) were calculated *via* the peak value of each metabolite. These metabolites produced in response to SA treatments were compared with the control values the difference in metabolites and fold change were analyzed after filtering with |log2(fold change (FC)) |˃1 and a significance *P* value ˂ 0.05.

### RNA extraction, RNA-seq, and miRNA-seq

The total RNA of each sample was extracted according to the RNAprep Pure Plant Kit manual (Tiangen Biotech Co., Beijing, China). A Nanodrop 2000 (Thermo Scientific, USA) and gel electrophoresis for RNA-seq and mRNA-seq analysis verified the RNA quality and integrity. All RNA samples were reverse transcribed into cDNA as reaction templates according to the QuantScript RT Kit and miRcute Plus miRNA First-Strand cDNA Kit manual (Tiangen Biotech Co. LTD, Beijing, China). The RNA-seq analysis steps were described as follows, briefly. The RNA-seq library was prepared following the TruSeq™ RNA Sample Preparation Kit from Illumina (San Diego, CA, USA) using 1 µg of total RNA. The messenger RNA was isolated and synthesized using a SuperScript double-stranded cDNA synthesis kit (Invitrogen, CA, USA) with random hexamer primers. Then, the synthesized cDNA was subjected to end repair, phosphorylation and ‘A’ base addition. The insert cDNA fragments of 300 bp were used for sequencing with the Illumina HiSeq X ten/NovaSeq 6000 sequencer (2 × 150 bp read length). With default parameters, the raw paired-end reads were trimmed and quality-controlled by SeqPrep and Sickle (https://github.com/najoshi/sickle ). The clean reads were separately aligned to the reference genome (https://www.ncbi.nlm.nih.gov/genome/?term=foxtail+millet ) using HIASAT software (https://ccd.jhu.edu/software/hisat2/index.shtml ). String Tie software was used to map assembled reads (https://ccb.jhu.edu/software/stringtie/index.shtml?t=example ). The DEGs between two samples were analyzed by DESeq software with default parameters, |log2(fold change (FC)) |˃1 and significance *P* value ˂ 0.05.

The miRNA-seq analysis was carried out as follows: 1) A small RNA library was generated by NEBNext Multiplex Small RNA library Prep Set for Illumina (NEB, USA) following the manual. 2) The size of insert cDNA fragments with 3’ and 5’ adaptors was 140~160 bp. 3) The library quality was assessed using DNA High Sensitivity Chips by Agilent Bioanalyzer 2100 system. 4) Illumina HiSeq 2500/2000 platform performed library sequencing, and 75 bp single-end reads were generated. 5) All identical sequences of sizes ranging from 18 to 32 nt were counted and used for a BLAST search of the Rfam database (https://rfam.sanger.ac.uk/ ) to remove non-miRNA sequences (rRNA, tRNA, snoRNA, etc.). Bowtie (http://bowtie-bio.sourceforge.net/index.shtml ) was used to annotate the chromosomal location against the reference genome data. Differentially expressed miRNAs (DEMs) were analyzed by BLAST searching the miRBase. The novel miRNAs were predicted by analyzing the characteristics of the hairpin structure of the miRNA precursor using miRDeep2 software (Kang et al., 2018). Using the DEseq2 software, significantly differentially expressed miRNAs were identified with |log2(fold change (FC)) |˃1 and a significance *P* value ˂ 0.05. Each miRNA target was predicted by using the tool psRNATarget (http://plantgrn.noble.org/psRNATarget/ ) with default parameters.

### qRT−PCR analysis

All gene-specific primers were designed by the NCBI online Primer-BLAST tools (www.ncbi.nlm.nih.gov/tools/primer-blast/ ) (**Table S1**, **S2**). Real-time quantitative PCR (qRT-PCR) analysis was carried out on a C1000 Touch™ deep-well thermal cycler (Bio-Rad, Hercules, CA, USA). One microgram of cDNA derived from mRNA and miRNA was used for qRT−PCR analysis according to the Quant qRT−PCR (SYBR Green) and miRcute miRNA (SYBR Green) Kit manuals. The mRNA and miRNA qRT−PCR reaction system was performed as follows: 1 µg of diluted cDNA was added as a template to each reaction system, which contained 1 µL of each reverse and forward gene-specific primer (10 µM), 10 µL of 2× TransStart Green qPCR Super Mix, and ddH_2_O to bring the total volume to 20 µL. The 2^-ΔΔCT^ method was used to calculate the expression level of each gene with reference genes *Actin* and *U6* (**Table S1** and **S2**) (Livak and Schmittgen, 2001). Three biological replicates were performed for each gene.

### Data deposition and analysis

All RNA-seq raw data were deposited in the NCBI database (accession number: PRJNA865014). All assays were repeated at least three times on three independent biological replicates. The data were collected and analyzed by Excel version 2019. The histograms were generated by the ggplot2 package of R (http://www.r-project.org, accessed in 2019). The significant difference between groups was determined using a one-way analysis of variance (ANOVA). KEGG enrichment analyses used the clusterProfiler package of R (Wu et al., 2021). The secondary structure of miRNA precursors was predicted by the RNAfold (http://rna.tbi.univie.ac.at/cgi-bin/RNAWebSuite/RNAfold.cgi ). The log2 value was used to normalize data with gene expression values and metabolites for cluster analysis and TBtools (Chen et al., 2020).

## Results

### Effects of SA treatment on folate content and metabolites in foxtail millet

**Figure 1:**
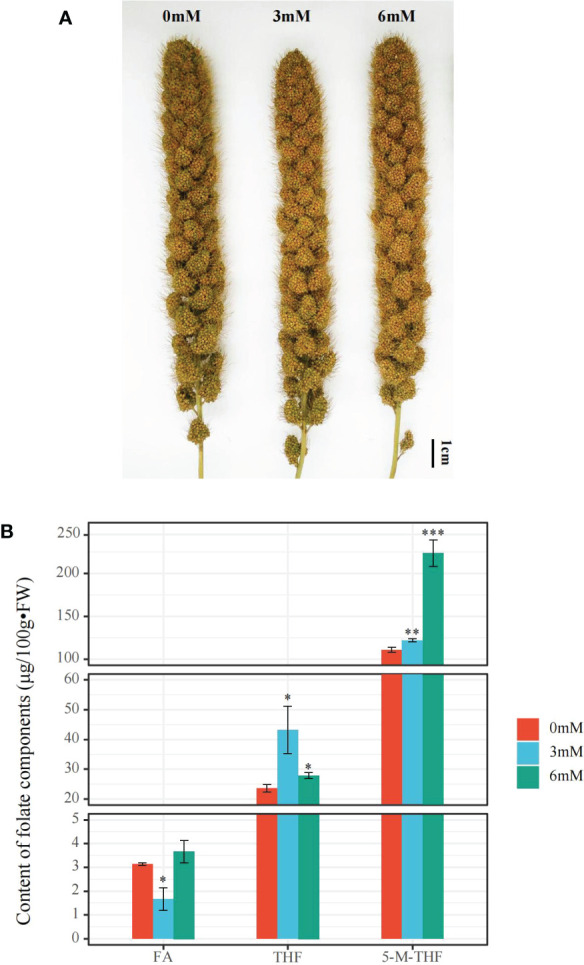
The phenotypic characteristics and folate content analyses in the panicle of foxtail millet treated with different concentrations of SA **(A)**: the phenotype of the panicle of foxtail millet with 0, 3, 6 mM SA treatment. **(B)**: the FA (Folate), THF (Tetrahydrofolate), and 5-M-THF (5-methyltetrahydrofolate) content of the panicle of foxtail millet with above SA treatment. “*” indicates significant difference (p < 0.05), “**” and “***” indicate extremely significant differences (p<0.01 and p<0.001).

After treatment with different SA concentrations, the total folate content of foxtail millet grains under SA treatment was significantly higher than that under the other SA treatments, reaching up to 255.6 μg/100g•FW after 6 mM treatment. Additionally, the 5-M-THF content under the 6 mM SA treatment was 1.83 and 2.01 times that under the 3 mM and 0 mM SA treatments, respectively. The THF content improved under the 3 mM SA treatment from 23.3 to 43 μg/100g•FW but did not significantly increase after the 6 mM treatment compared to the other SA treatments. The FA content, which was 3.7 μg/100g•FW under the 6 mM SA treatment, was higher than that under the 3 mM SA treatment (1.7 μg/100g•FW) (**Figure 1**).

An untargeted metabolome analysis was performed on B24 grains treated with different concentrations of SA. A total of 2604 positive ion peaks were detected, including 275 known metabolites. Principal component analysis revealed good stability and repeatability among different samples. The QC samples had no significant drift according to the principal component scores, which indicated that the data were reliable and repeatable (see **Figure S1**).

**Figure 2:**
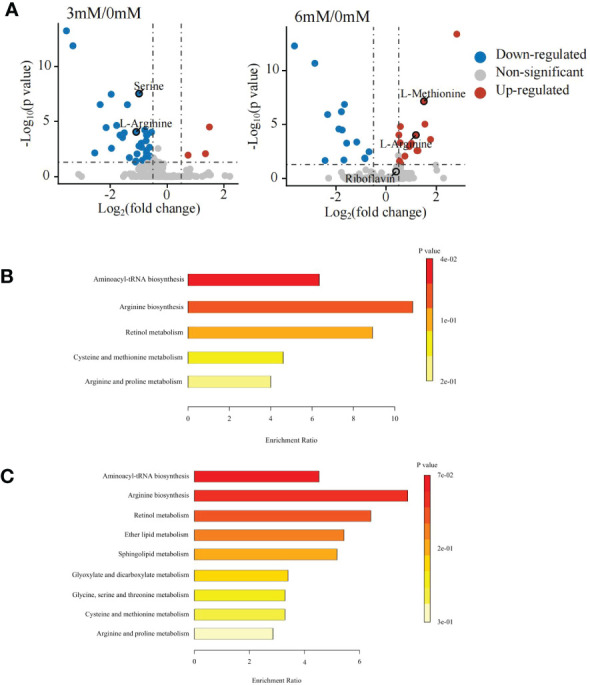
Differentially accumulated metabolites and KEGG enrichment analyses in the panicle of foxtail millet following SA treatment using untargeted metabolome **(A)** volcanic map of differential metabolites displayed by comparing 3 mM and 6 mM SA treatment to the control (CK); **(B)** the KEGG enrichment of differential metabolites analyzed by comparing 3mM SA treatment to CK; **(C)** the KEGG enrichment of differential metabolites analyzed by comparing 6mM SA treatment to CK.

The differential metabolite analysis showed that under positive ion mode, 36 differential metabolites were detected between the 0 mM and 3 mM SA treatments, of which 3 metabolites were up-regulated, and 33 were down-regulated. Under the 6 mM treatment, 31 different metabolites were determined, of which 17 metabolites were up-regulated and 14 were down-regulated (**Figure 2A**).

The KEGG enrichment analysis showed that the differential metabolites in the 3 mM SA treatment were enriched in aminoacyl-tRNA biosynthesis, arginine biosynthesis, retinol metabolism, cysteine, methionine, arginine and proline metabolic pathways when compared to the 0 mM SA treatment (**Figure 2B**). However, the 6 mM SA treatment showed a greater abundance of metabolites enriched in 9 metabolic pathways, compared with the control, including aminoacyl-tRNA biosynthesis, arginine biosynthesis, retinol metabolism, ether lipid metabolism, sphingolipid metabolism, glyoxylate and dicarboxylate metabolism, glycine metabolism, serine and threonine metabolism pathways (**Figure 2C**).

The relative contents of Arg, Met, Pro, Ser and Riboflavin were further analyzed (**Figure S2**). Comparing the 3 mM and 6 mM SA treatments to the control, the results showed that Arg, Met and Pro contents were significantly increased in response to 6 mM SA treatment but significantly decreased after the 3 mM SA treatment. Primarily, the Met content after 6 mM SA treatment was 3.14 times greater than the control, whereas after 3 mM SA treatment it was significantly lower (0.68-fold) than that of the control. Meanwhile, the Ser content was reduced in response to both the 3 mM and 6 mM SA treatments. In addition, the riboflavin content was significantly increased by treatment with the two different SA concentrations, up to 1.53-fold.

### Differentially expressed gene analysis

After SA treatment, comparative transcriptome analysis was performed to explore the differentially expressed genes (DEGs). In total, 66.26 Gb of clean data and 118,664,398 clean reads were obtained from the RNA-Seq sequencing of all samples. A total of 44.05 Mb of raw reads were obtained from miRNA sequencing, and 10,581,629 and 10,238,530 clean reads were obtained from the control and 6 mM SA treatment samples, respectively. Each sample’s mRNA average Q30 base percentage was no less than 94.91%, while miRNA Q30 base percentage was no less than 96.10%, indicating a higher quality of sequencing data (**Table S3**, **S4**).

**Figure 3:**
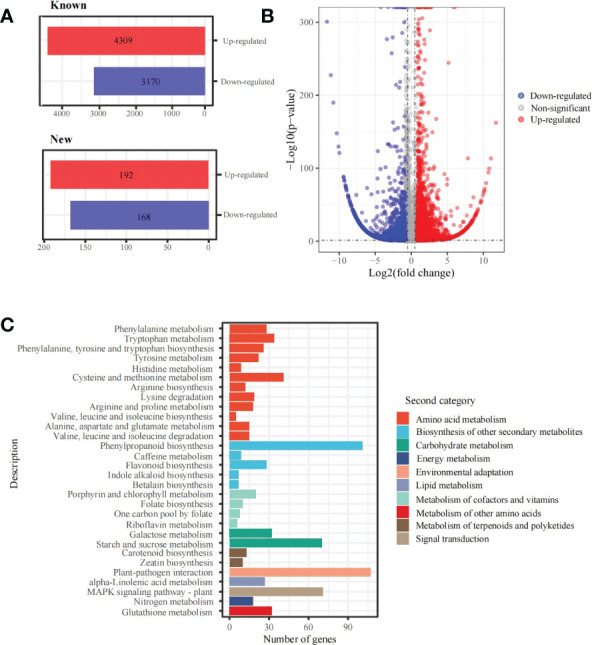
DEGs and KEGG enrichment analyses in the panicle of foxtail millet following SA treatment **(A)** the statistics of new and known DEGs; **(B)** volcano plot of DEGs between 6mM and 0 mM SA treatment; **(C)** the KEGG enrichment of DEGs.

In addition, 7,839 differentially expressed genes (DEGs) under the 6 mM SA treatment were identified compared with the control. There were 4,501 up-regulated and 3,338 down-regulated transcripts. Among them, 4,309 up-regulated and 3,170 down-regulated genes were annotated with known functions and 192 up-regulated and 168 down-regulated genes were new predictions (**Figures 3A, B**). The KEGG functional enrichment analysis showed that these DEGs were associated with 131 metabolic pathways (**Figure 3C**). For folate biosynthesis (KO00790), folate-dependent 1C metabolic and riboflavin metabolic (KO00740) pathways, there were 13 DEGs, including *HPPK/DHPS* (folate synthesis bi-functional protein), *MOCS1A/1B* (GTP 3’,8-cyclase), *FTHFD* (formyltetrahydrofolate deformylase), *MTRF* (methionyl-tRNA formyltransferase), *5FCL1/2* (5-formyltetrahydrofolate cyclo-ligase), *DHFR1/2* (dihydrofolate reductase), *RIBA1/2* (bi-functional riboflavin biosynthesis protein), *PYRP2* (5-amino-6-(5-phospho-D-ribitylamino)uracil phosphatase) and *RFK* (bi-functional riboflavin kinase). In addition, the KEGG enrichment and metabolome analysis also showed that the DEGs were enriched in the amino acid metabolic pathway. For the Met metabolic pathway, there were 15 DEGs, including *CYSC1*, *CYSK* (cysteine synthase), *AK* (bifunctional aspartokinase/homoserine dehydrogenase), *BHMT2* (homocysteine S-methyltransferase), *metE* (5-methyltetrahydropteroyltriglutamate–homocysteine methyltransferase), *DNMT1* (DNA (cytosine-5)-methyltransferase), *ACS* (1-aminocyclopropane-1-carboxylate synthase), *AMD1* (S-adenosylmethionine decarboxylase), *SRM* (S-adenosylmethionine synthetase), *ACO1/2* (aminocyclopropanecarboxylate oxidase), *APIP* (bi-functional methylthioribulose-1-phosphate dehydratase/enolase-phosphatase), *ADI1* (1,2-dihydroxy-3-keto-5-methylthiopentene dioxygenase) and *TAT1/2* (tyrosine aminotransferase).

### Analysis of DEGs involved in the folate and Met metabolism pathways

**Figure 4:**
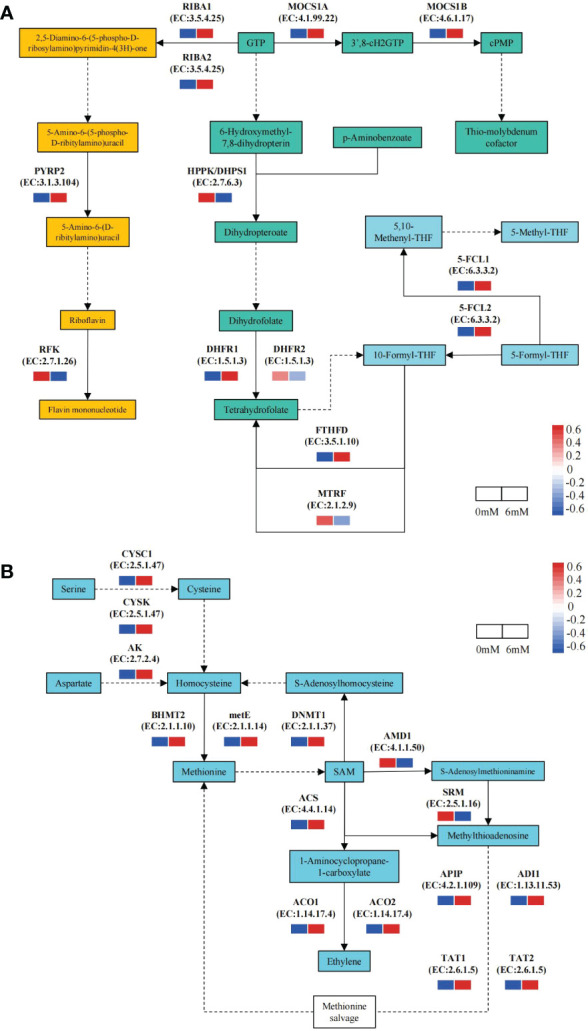
The expression pattern of DEGs related to the folate and Met metabolic pathway the squares represent the metabolites in the folate metabolism pathway; Yellow background indicates genes in the riboflavin metabolic pathway, green background indicates folate synthesis pathway genes; blue background shows folate-dependent 1C metabolism pathway; solid and dotted line arrow indicated directly and indirectly products synthesis route by downstream enzyme and substances. **(A)**: PYRP2: 5-amino-6-(5-phospho-D-ribitylamino)uracil phosphatase; RFK: bi-functional riboflavin kinase; RIBA1/RIBA2;bi-functional riboflavin biosynthesis protein; MOCS1A/1B: GTP 3’,8-cyclase; HPPK/DHPS1: folate synthesis bi-functional protein; DHFR1/DHFR2: dihydrofolate reductase; FTHFD: formyltetrahydrofolate deformylase; MTRF: methionyl-tRNA formyltransferase; 5-FCL1/5-FCL2: 5-formyltetrahydrofolate cyclo-ligase. **(B)**:CYSC1/CYSK: cysteine synthase; AK: bi-functional aspartokinase/homoserine dehydrogenase; BHMT2: homocysteine S-methyltransferase; metE: 5-methyltetrahydropteroyltriglutamate-homocysteine methyltransferase; DNMT1: DNA (cytosine-5)-methyltransferase; ACS: 1-aminocyclopropane-1-carboxylate synthase; ACO1/2: 1-aminocyclopropane-1-carboxylate oxidase; AMD1: S-adenosylmethionine decarboxylase proenzyme; SRM: spermine synthase; APIP: bi-functional methylthioribulose-1-phosphate dehydratase/enolase-phosphatase; ADI1: 1,2-dihydroxy-3-keto-5-methylthiopentene dioxygenase; TAT1/TAT2: tyrosine aminotransferase.

Focusing on folate metabolism pathway, 13 DEGs were identified by comparing the 6 mM SA treatment to the 0 mM SA treatment. Of these, 9 DEGs were up-regulated in the 6 mM SA treatment, 1.35- to 3.74-fold higher than the control. In the folate biosynthesis pathway, the expression level of *DHFR1* was up-regulated, suggesting that it can promote THF accumulation. In the folate-dependent 1C metabolism pathway, the up-regulated expression levels of *5-FCL1/2* and *FTHFD* promoted 5-M-THF synthesis. In the riboflavin metabolic pathway, except for *RFK*, the DEG expression levels in the 6 mM SA treatment were higher than in the control (**Figure 4A**).

Moreover, there were 15 DEGs related to the Met metabolism pathway, of which 13 were up-regulated in response to the 6 mM SA treatment, and the transcript levels were 2.03- to 9.33-fold higher than in the control (**Figure 4B**). The expression levels of the *CYSC1* and *CYSK* genes involved in the Ser to homocysteine (Hcy) transformation pathway and the *AK* gene involved in the Asp to Hcy transformation pathway were also enhanced. The expression levels of the *BHMT2* and *metE* genes, which play an essential role in Met synthesis by Hcy, and the *DNMT1* gene, which is involved in the Hcy synthesis pathway by Met feedback, also increased. In the ethylene biosynthesis and Met salvage pathway (Yang Cycle), the expression levels of the *ACS*, *ACO1/2*, *ADI1* and *TAT1/2* genes increased compared with those in control.

### Identifying and predicting miRNAs targeted genes related to the folate and Met metabolic pathways

**Figure 5:**
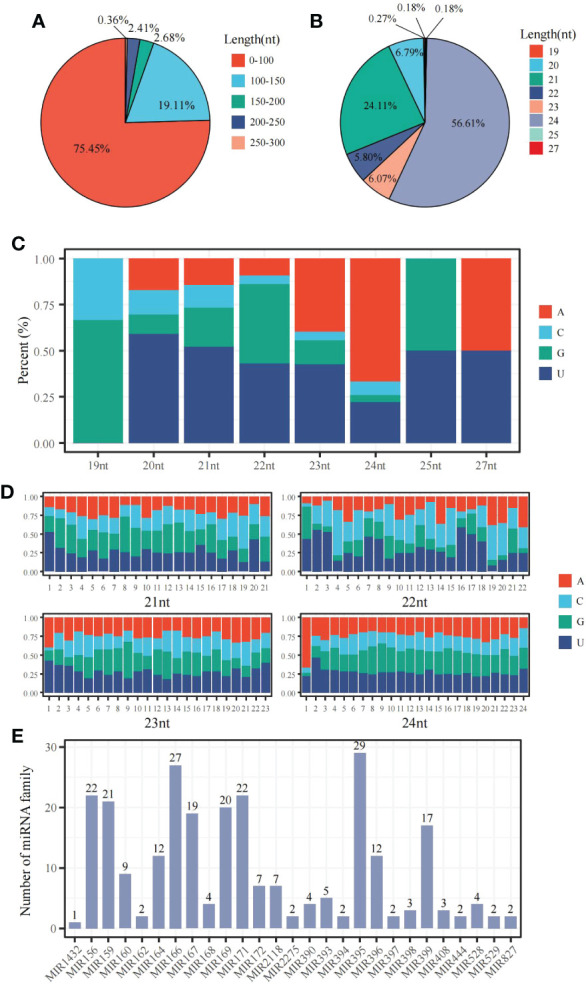
The distribution characteristic of miRNAs in the panicle of foxtail millet following SA treatment **(A)** the sequence length distribution of pre-miRNA; **(B)** the sequence length distribution of mature-miRNA;**(C)** the base bias analysis of miRNA at the first nucleotide position; **(D)** the bias analysis of each base for 21~24 nt miRNA; **(E)** the distribution of miRNA family members.

The miRNA-sequencing analysis showed that 233 known and 347 new miRNAs were detected following 6 mM SA treatment. In the 0 mM SA treatment, 229 knowns and 410 new miRNAs were also detected. By comparing the 6 mM SA treatment to the 0 mM SA treatment, a total of 367 miRNAs were differentially expressed, including 104 down-regulated and 263 up-regulated miRNAs. Meanwhile, the characteristic nucleotide number of miRNA showed that pre-miRNAs had less than 100 nt and accounted for 75.45% of the total, and those with 100~150 accounted for 19.11% of the total (**Figure 5A**). Most mature miRNAs were 21 and 24 nt, accounting for 56.61% and 24.11% of the total, respectively (**Figure 5B**). The nucleotide bias preferred U at the first position of the miRNA base for the 21~24 nt miRNA (**Figure 5C**). A conserved nucleotide bias analysis revealed that the ninth position of these 21~23 nt miRNAs was G/C, which accounted for 60%~72.3%. For the 24 nt miRNA, the conserved nt bias was G/C at the eighth position, accounting for 57.1% (**Figure 5D**). A total of 262 miRNAs were classified into 27 miRNA families. In addition, the distribution characteristics of the miRNA family showed that the miRNA395 and miRNA166 families, containing 29 and 27 members, respectively (**Figure 5E**) were among the most significant.

**Figure 6:**
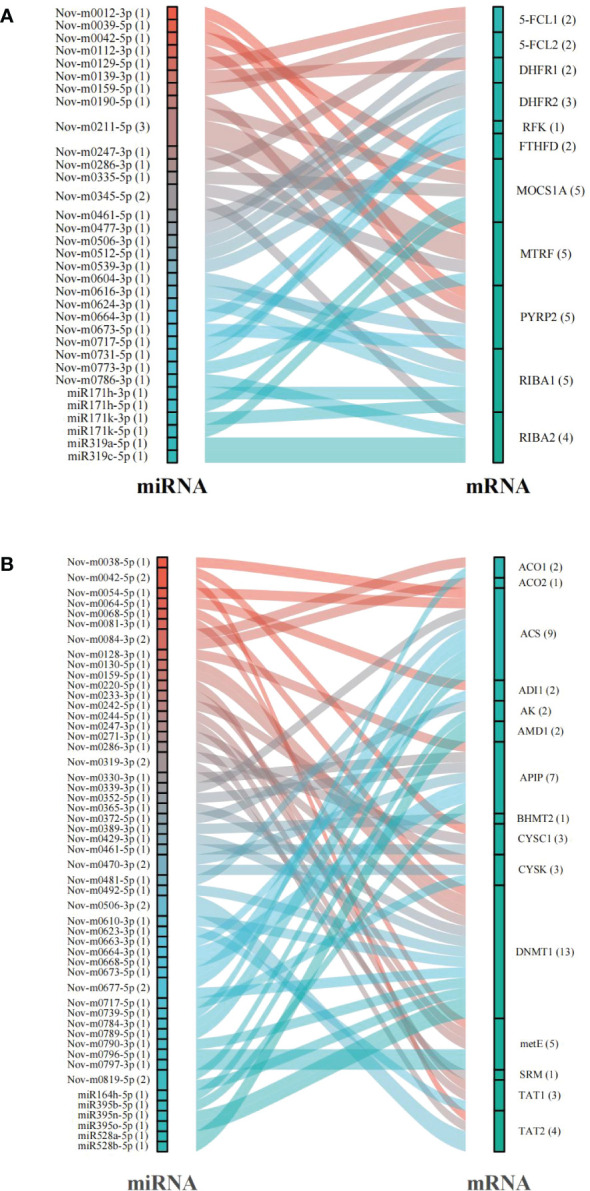
The prediction of miRNAs targeted mRNAs related to folate **(A)** and Met **(B)** metabolism pathway.

An *in silico* prediction showed that a total of 119 miRNAs targeted 13 DEGs related to folate metabolism and formed 129 miRNA−mRNA interaction combinations. Thirty-three of these miRNAs, including 18 that were up-regulated and 15 that were down-regulated under the 6 mM SA treatment, were identified as likely to target 11 DEGs, and 36 miRNA−mRNA interaction combinations were obtained. The results showed that Nov-m0211-5p regulated the target genes *MOCS1A* and *MTRF* and Nov-m0345-5p regulated the target genes *MTRF* and *RIBA2*. Moreover, we found that *MOCS1A*, *MTRF*, *PYRP2*, and *RIBA1* were regulated by 5 miRNAs, while one miRNA (Nov-m0664-3p) targeted the RFK gene (**Figure 6A**; **Table S5**).

In addition, 15 mRNAs were predicted to be regulated by 179 miRNAs in the Met metabolic pathway. 58 miRNA−mRNA interactions related to the Met metabolic pathway contained 32 up-regulated and 19 down-regulated miRNAs. Especially, *ACS* and *DNMT1* genes were regulated by 9 and 13 miRNAs, respectively (**Figure 6B**; **Table S6**). *CYSC1*, a key gene catalyzing the Ser/Cys exchange, was regulated by Nov-m0319-3p, Nov-m0416-5p and Nov-m0042-5p.

**Figure 7:**
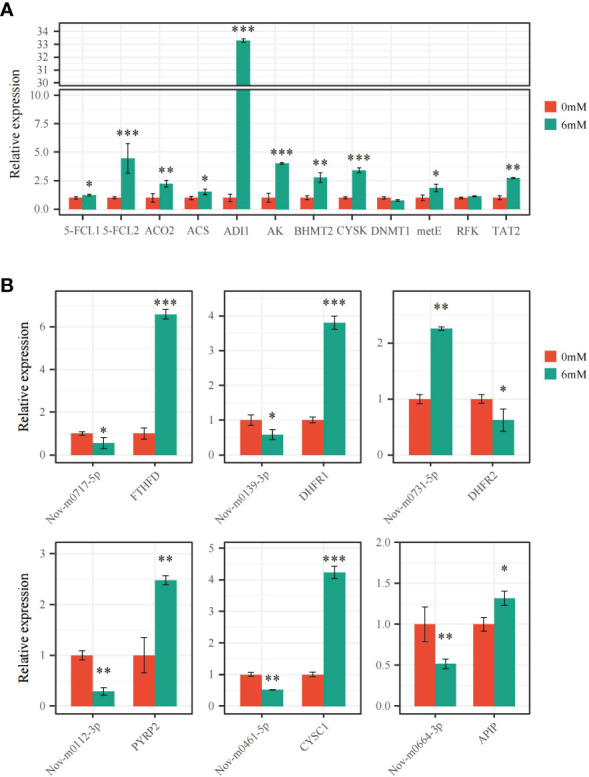
qRT-PCR verification of the expression pattern of miRNAs and DEGs **(A)** the expression level of DEGs related to folate and Met metabolism pathway were analyzed by qRT-PCR; **(B)** the expression level of miRNAs and targeted FTHFD, DHFR1/2, PYRP2, CYSC1 and APIP genes were analyzed by qRT-PCR. “*” indicates significant difference (p < 0.05), “**” and “***” indicate extremely significant differences (p<0.01 and p<0.001).

The expression levels of 6 miRNAs and 18 target genes were analyzed by qRT−PCR to verify differentially expressed miRNAs and target mRNAs. Following the 6 mM SA treatment, *5-FCL1/2*, *FTHFD*, *DHFR1*, and *PYRP2* expression levels significantly increased compared to the control, and the *FTHFD* and *5-FCL2* expression levels were up-regulated 6.69- and 4.47-fold, respectively, compared with the CK. For the Met metabolic pathway, 10 DEGs were significantly up-regulated by 1.32~33.29-fold under the 6 mM SA treatment compared with the CK. However, the expression level of *DNMT1* was down-regulated 0.77 times compared with the CK. All gene expression levels were consistent with transcriptomic data analysis results, except for the *RFK* and *DNMT1* genes (**Figure 7A**). The correlation coefficient of the FPKM value and relative expression level of these genes was up to 0.92 (**Figure S3A**).

The secondary structure of the precursor miRNAs was predicted for Nov-m0717-5p, Nov-m0139-3p, Nov-m0112-3p, Nov-m0461-5p, Nov-m0664-3p, and Nov-m0731-5p, showing that they can form a stable stem-loop structure (**Figure S4**). The qRT−PCR analysis of these miRNAs proved that their expression levels had the opposite trends as their target genes (**Figure 7B**). Nov-m0717-5p, Nov-m0139-3p, Nov-m0112-3p, Nov-m0461-5p, and Nov-m0664-3p were significantly down-regulated by 0.29~0.58-fold compared with the control (CK). However, the target expression levels for *FTHFD*, *DHFR1*, *PYRP2*, *CYSC1*, and *APIP* were significantly up-regulated 1.32~6.59-fold compared with the CK. The miRNA expression pattern showed the same trend as the transcriptomic analysis results. The correlation coefficient between these miRNAs’ FPKM value and the relative expression level was up to 0.85 (**Figure S3B**).

## Discussion

### Exogenous SA enhanced the folate and methionine metabolism and regulated relative synthesis genes in foxtail millet

In plants, salicylic acid serves to stimulate plant secondary metabolism and transcriptional reprogramming, resulting in redirecting metabolic flux towards secondary metabolite production (Khan et al., 2015). SA is known to improve abiotic stress tolerance by regulating the production of major plant metabolites, such as anthocyanins, furanocoumarins, alkaloids, glucosinolates and phenolics (Rodas-Junco et al., 2013; Khan et al., 2015). Previously, a few research reports have shown that SA is a key phytohormone that enhances the folate content and expression level of genes related to the folate biosynthesis pathway in Arabidopsis and coriander (Puthusseri et al., 2013; Puthusseri et al., 2018). Our study found that 3 mM and 6 mM SA treatment of the panicle of foxtail millet also enhanced the folate content, especially the accumulation of 5-M-THF. In maize and potato studies, the expression level of some key genes like *ADCS*, *DHFR*, *5-FCL* and *GGH* could be responsible for folate accumulation (Lian et al., 2015; De Lepeleire et al., 2018). Our previous study reported that the expression level of *ADCL1*, *GGH* and *SHMT3* genes is correlated with folate accumulation in the panicle development stage of foxtail millet (Hou et al, 2022a). These results suggested that folate accumulation was mainly correlated with these key genes involved in folate synthesis and 1C metabolism. Here, we report a comparative transcriptome analysis of panicles treated with different concentrations of SA in foxtail millet. Furthermore, we found that folate synthesis-related gene expression could be regulated by SA. Surprisingly, *DHFR1*, *FTHFD*, *MTRF*, and *5-FCL1/2*, which are involved in the folate metabolic pathway, were more highly expressed in the 6 mM SA treatment than in the control (CK), and transcript levels of the *metE* and *BHMT2* genes, which are involved in the Met synthesis pathway. In addition, we also found that the *ACS*, *ACO1/2*, *AMD1*, *SRM*, *APIP*, *ADI1* and *TAT1/2* genes were more highly expressed in the ethylene biosynthesis and Met resynthesis pathway in response to SA treatment. This suggested that Met and folate accumulation induced by SA in the panicle of foxtail millet may be related. Folates, as one carbon donors, participate in methyl transfer reactions in plants related to epigenetic regulation by S-adenosyl-methionine synthetase (Gonzalez and Vera, 2019). In Arabidopsis, a proteomic analysis of the effect of SA on seed germination revealed that it affected Met metabolism by inducing the expression of S-adenosyl-Met synthetase and S-adenosylhomo-Cys hydrolase (Rajjou et al., 2006). In rice, exogenous SA alleviated chromium (Cr) stress in the roots and elevated metabolites, including cysteine, methionine, glutathione, proline, etc. (Huda et al., 2016). In maize roots, treatment with SA enhanced methionine content and improved Pb stress tolerance (Zanganeh et al., 2020). These results support our observation that metabolites concentrations change in the panicle of foxtail millet treated with exogenous SA treatment.

### SA mediated miRNAs regulate the key genes related to folate and Met metabolic pathways

**Figure 8:**
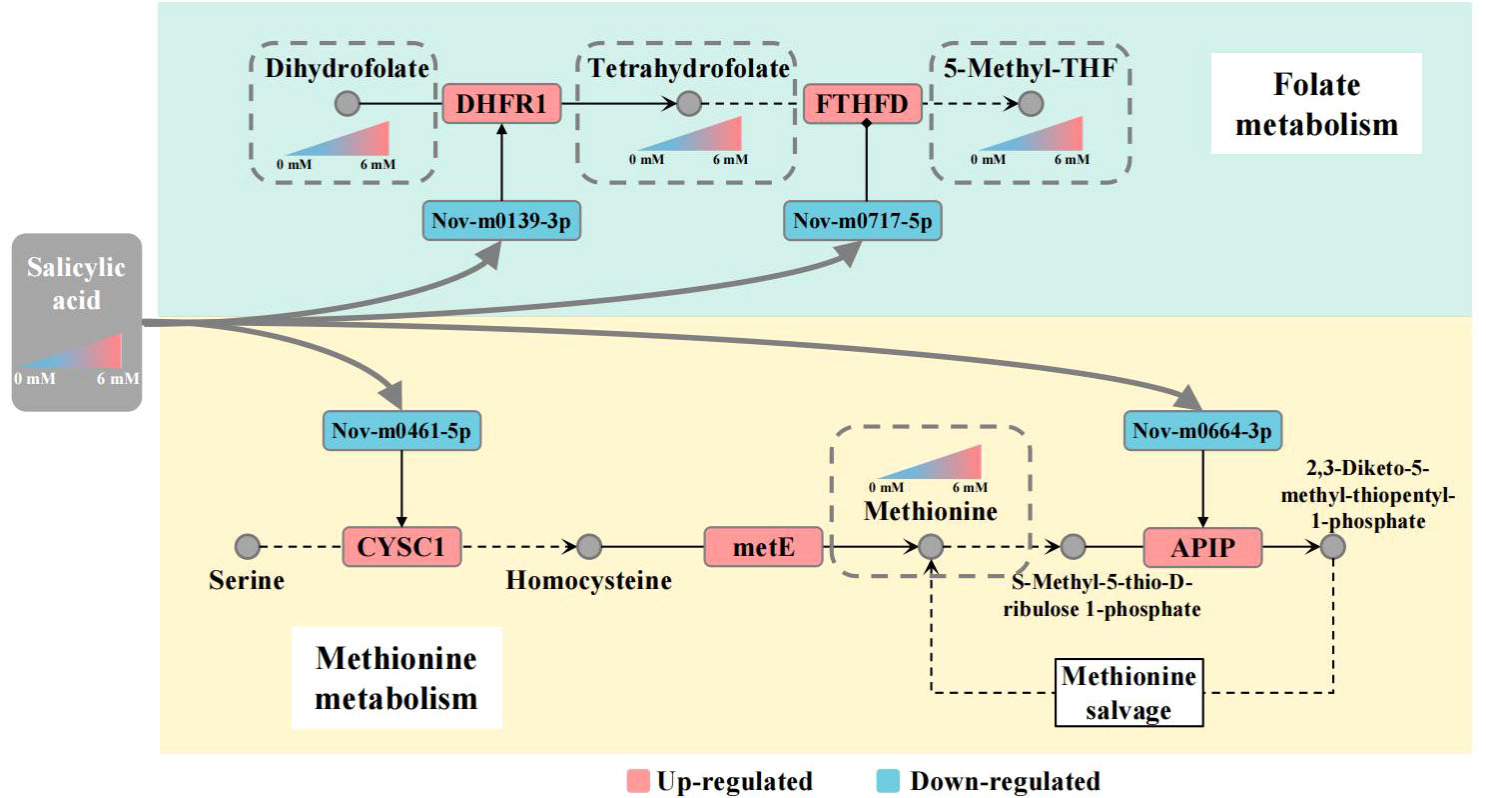
Working model for salicylic acid-mediated miRNA regulation of folate and Met metabolism Note: The gray nodes represent metabolites, the squares represent mRNA/miRNA, and the color of the squares represents the up-regulated or down-regulated of the transcript level of mRNA/miRNA; The metabolites are connected by black arrows, and the dotted line indicates that multiple metabolic processes are omitted.

Previously, a few reports have indicated that SA is a key signal molecule regulating plant innate immunity *via* the folate-dependent metabolism pathway. In Arabidopsis, application of SA and folate precursors 7,8-dihydroneopterin (DHN) enhanced resistance to *P. syringae*. The elevated transcript level of SA marker gene *PATHOGENESIS-RELATED1* suggests that folate induces local and systemic SA-mediated defense (Wittek et al., 2015). Recent findings indicate that epigenetic regulation of gene expression is controlled by folate-dependent one-carbon metabolism using methyltransferase (METS1) to methylate DNA and histones (Gonzalez and Vera, 2019). In Met salvage or Yang cycle pathway, 5-M-THF as methyl donor took part in the process of transforming homocysteine to methionine (García-Salinas et al., 2016). These results suggest that SA is linked with folate and Met metabolism by regulating transcript levels of some key genes regulators in plants. In our study, we observed that SA enhanced folate and Met accumulation was accompanied by elevated transcript levels of *FTHFD*, *DHFR*, *CYSC1* and *APIP* genes in panicles of foxtail millet. However, the post-transcriptional regulatory mechanism of folate and Met metabolism in plants is poorly understood. MicroRNAs, as posttranscriptional gene regulators, play important roles in plant development, abiotic stress tolerance and biotic stress (Jones-Rhoades et al., 2006; Owusu et al., 2021). For foxtail millet, many miRNAs and their targets have been identified and verified by miRNA-seq analysis in different varieties, tissues, and under drought stress conditions (Yi et al., 2013; Deng et al., 2022). We found 347 and 410 new miRNAs, which was more than previously reported and this might be explained by the use of different varieties, tissues and treatments. In particular, we found 33 and 51 new miRNAs targeted 11 and 15 genes, respectively, involved in the folate and Met metabolic pathways. In addition, 6 new miRNA-mRNA interaction related to regulate folate and Met metabolism were verified by qRT-PCR. However, these miRNA expression level were significant negative correlation with mRNAs expression level. Meantime, we also predicted that these target mRNAs in nucleotide sequence are cleaved within miRNA-binding site. The results indicated that these miRNAs regulated the expression of target mRNAs by degradation model. Here, we suggest that SA is linked with folate and Met metabolism pathways through the up-regulating *DHFR1*, *FTHFD*, *CYSC1* and *APIP* gene expression level by down-regulating expression of Nov-m0139-3p, Nov-m0717-5p, Nov-m0461-5p and Nov-m0664-3p. Furthermore, some researches had reported that miRNA production could be modulated by DNA methylation and other epigenetic mechanisms directly, or indirectly affecting the signal pathway. In humans, miRNA-22-3p and miRNA-149-5p are differentially expressed in normal and cancerous human hepatocytes, and regulate *DHFR* transcript level resulting in folate deficiency (Li et al., 2017). Meantime, altered miRNAs expression could be associated with low folate status in pregnant women (Beckett et al., 2017). Our results suggest that exogenous SA acts as a signal molecule enhancing the folate and Met content in the panicle of foxtail millet. However, the DEG and DEM analyses revealed a complex regulatory network between mRNAs and miRNAs involved in folate and Met metabolism pathways under SA treatment in foxtail millet. We considered that there might be a potential molecular regulatory mechanism linking SA-mediated miRNA and folate and Met accumulation in foxtail millet. We propose such a potential gene regulatory network in the **Figure 8**. In response to SA treatment, Nov-m0139-3p and Nov-m0717-5p miRNAs up-regulate *DHFR1* and *FTHFD* gene expressed level lead to increase the THF and 5-M-THF content in the panicle of foxtail millet. Nov-m0461-5p and Nov-m0664-3p up-regulate *CYCS1* and *APIP* gene expression level, elevating the Met content in the panicle of foxtail millet. We anticipate that these results will help elucidate the miRNA−mRNA regulatory mechanism of folate and methionine accumulation in foxtail millet with SA treatment and identify new gene targets for genetically improving folate content in the future.

## Data availability statement

The datasets presented in this study can be found in online repositories. The name of the repository and accession number can be found below: NCBI Sequence Read Archive; PRJNA865014.

## Author contributions

SH and YM: data curation, formal analysis, methodology, and writing original draft. YZ, GM and YM: data curation, formal analysis, and methodology. YH, HL, and KZ: data curation, funding acquisition, and investigation. ZS: conceptualization, investigation, project administration, resources, supervision, writing review and editing, and first corresponding author. All authors contributed to the article and approved the submitted version.

## Supplementary material

Supplementary Figure 1 The principal component (PCA) analysis of metabolites in the panicle of foxtail millet with SA treatment

Supplementary Figure 2 The relative content of differential metabolites in foxtail millet panicle with SA treatment Note: “*” indicates significant difference (P < 0.05), “**” and “***” indicate extremely significant differences (p<0.01 and p<0.001).

Supplementary Figure 3 The correlation analysis of gene expression level between FPKM and qRT-PCR values (A): the correlation analysis between FPKM and qRT-PCR value of DEGs; (B): the correlation analysis between FPKM and qRT-PCR value of miRNAs.

Supplementary Figure 4 The second structure prediction of miRNAs targeted genes related to folate and Met pathway

Supplementary File 1 The metabolic profiling data of the panicle with SA treatment in foxtail millet
