# Supplementary figures and images for: Role of miRNAs in regulation of SA-mediated upregulation of genes involved in folate and methionine metabolism in foxtail millet
Source: Front Plant Sci. 2022 Dec 6;13:1023764. doi: 10.3389/fpls.2022.1023764 (PMC9763449; doi:10.3389/fpls.2022.1023764)

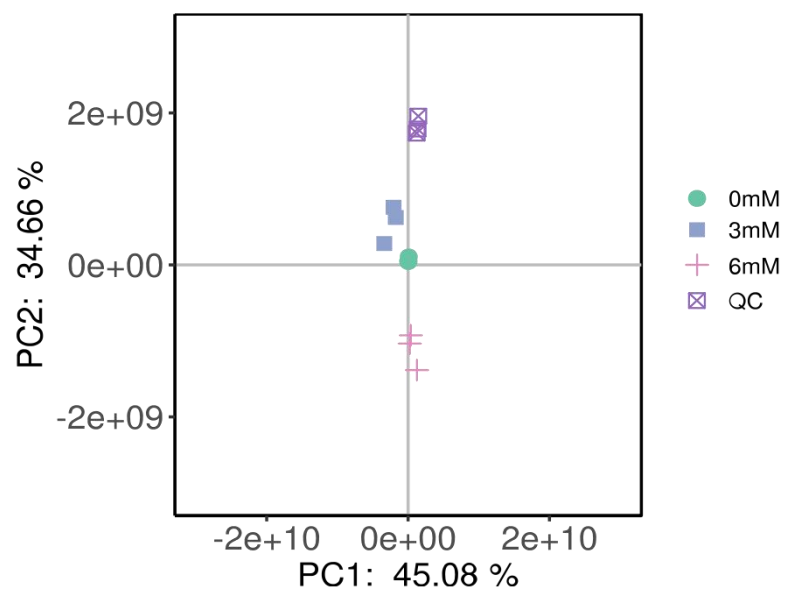

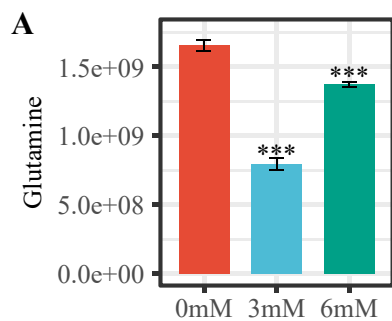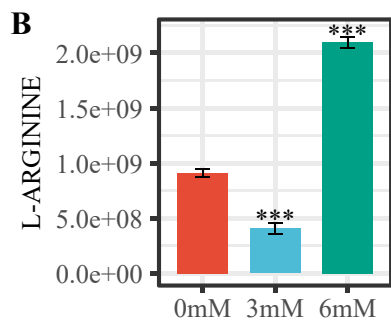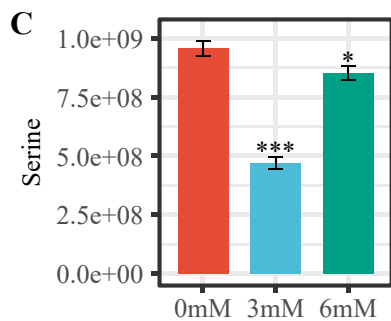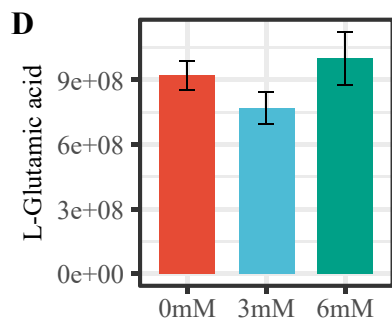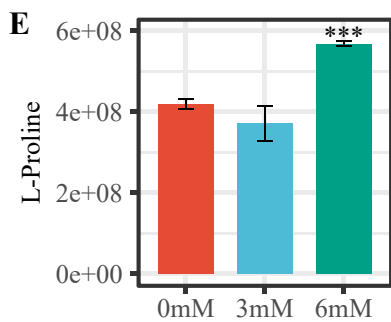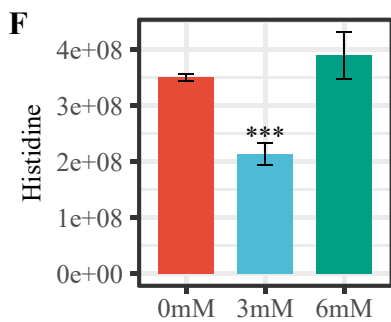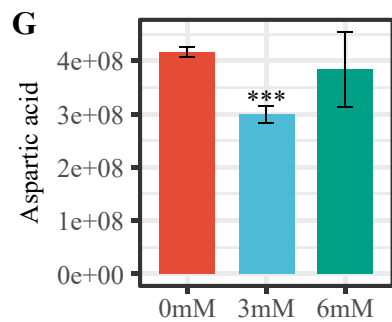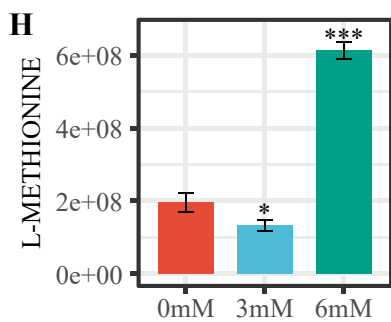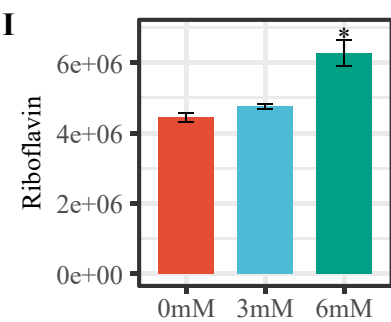

**A**

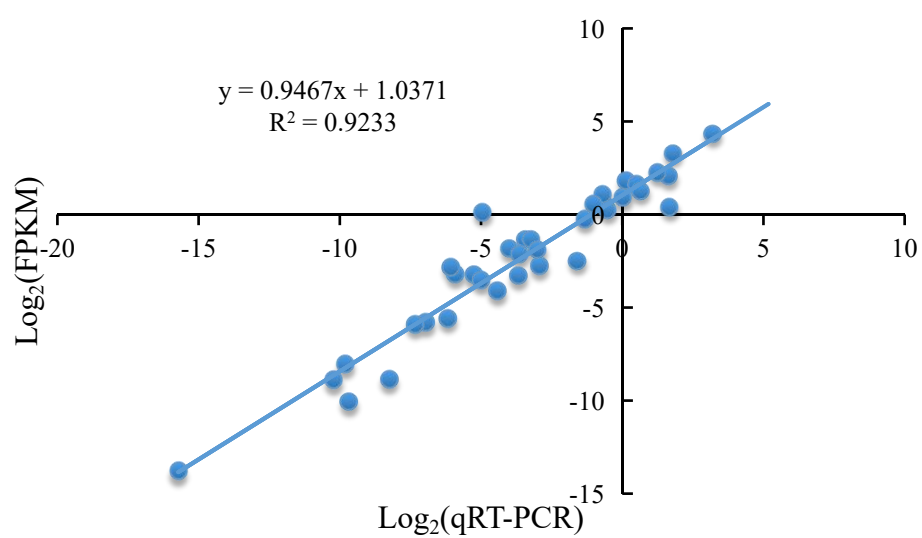

**B**

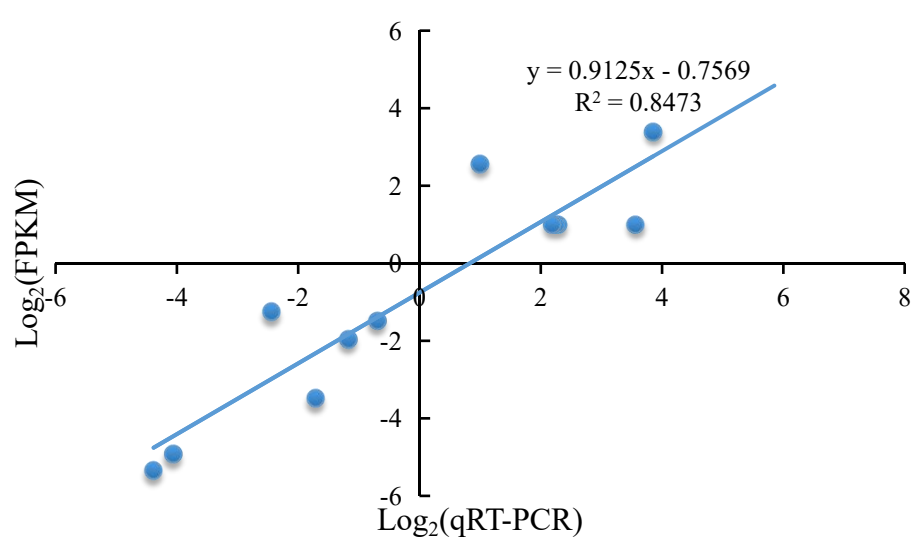

Nov-m0717-5p

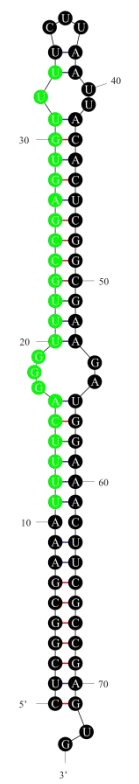

Nov-m0112-3p

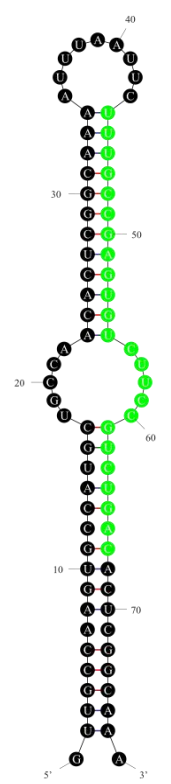

Nov-m0461-5p

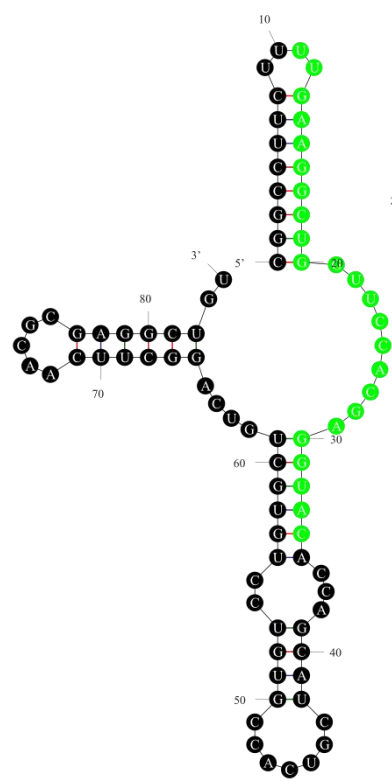

Nov-m0664-3p

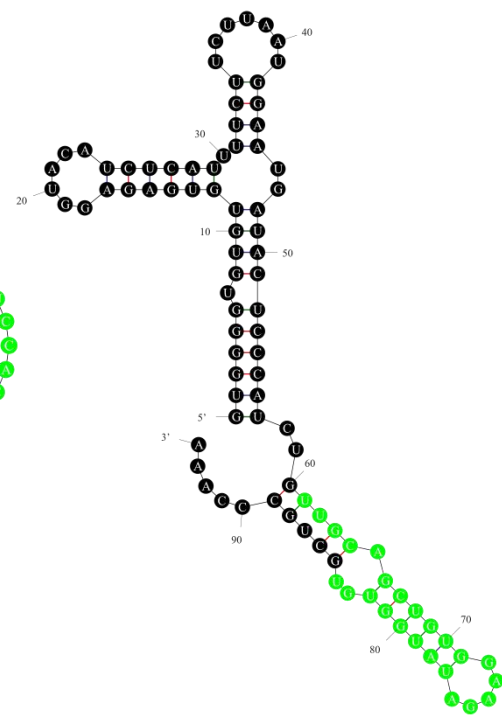

Nov-m0731-5p

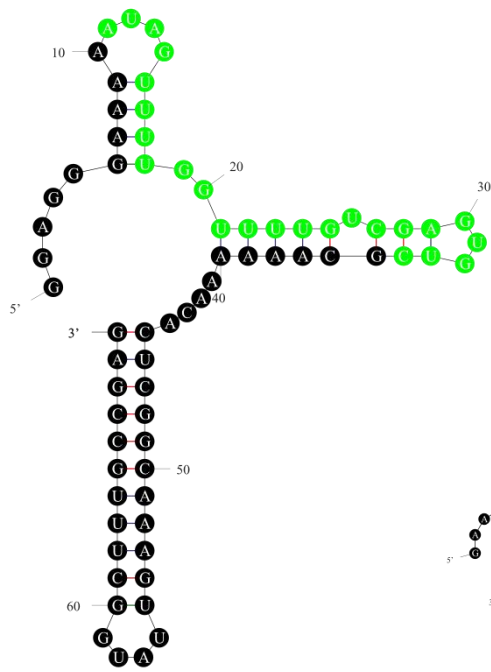

Nov-m0139-3p

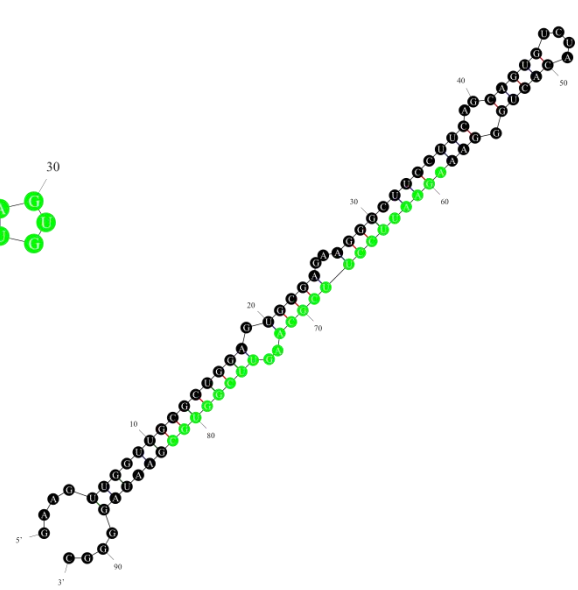

Supplement: Supplementary Figure 1 — The principal component (PCA) analysis of metabolites in the panicle of foxtail millet with SA treatment [file Image_1.pdf]
