# Supplementary material for: Role of miRNAs in regulation of SA-mediated upregulation of genes involved in folate and methionine metabolism in foxtail millet
Source: Front Plant Sci. 2022 Dec 6;13:1023764. doi: 10.3389/fpls.2022.1023764 (PMC9763449; doi:10.3389/fpls.2022.1023764)
Supplement: Supplementary file 3 [file Table_1.docx]

**Table S1 The qRT-PCR primers used in the study for miRNAs**

| miRNA | Sequence |
| --- | --- |
| U6 | GCCGCAAGGATGACACGC |
| Nov-m0461-5p | TTGAAGGCTGGTTCCACGAG |
| Nov-m0506-3p | CTACTCTTCGTCGCGGACTG |
| Nov-m0159-5p | GACGGAAGACACTCGGCAAA |
| Nov-m0673-5p | TTTGCAGCTGTGTGAAGTGAGT |
| Nov-m0664-3p | TTGCAGCTGTGGAAGATATG |
| Nov-m0717-5p | TTTCAGGGTTTGCCGAGTGT |
| Nov-m0139-3p | GCACTGGATACGACAGAATT |
| Nov-m0112-3p | GCACTGGATACGACTTTGCC |
| Nov-m0731-5p | GCACTGGATACGACATAGTT |

**Table S2 The qRT-PCR primers used in the study for DEGs**

| mRNA | Sequence | mRNA | Sequence |
| --- | --- | --- | --- |
| Siactin-F | TGCTCAGTGGAGGCTCAACA | ADI1-F | AAGTGGATTCGCGTAGCAGT |
| Siactin-R | GAGCGCAAGTACAGTGTCTGG | ADI1-R | GTCCAGATACTCCTGCCTCG |
| FTHFD-F | GCAGAACGTGTCGAAATGACC | AK-F | AAACCACACTGGCTGTTGGA |
| FTHFD-R | CGAAGCAATGGGACAGGTTG | AK-R | GCCGGTTCTGCTTCAGTTTG |
| 5-FCL1-F | GCCAGCGAAGATCTGGCTAT | APIP-F | AAGGCAACTGCTGTCCTTGT |
| 5-FCL1-R | TCGCAACTGCTGACAGCTTA | APIP-R | TTCAGCCTGTGTCTTGGCAT |
| 5-FCL2-F | CAACCTCGGCTAAGGACAGG | BHMT2-F | GAACGCAAGCGAGAAGGTTG |
| 5-FCL2-R | CTGCGAATCCCTCACCCTTC | BHMT2-R | CGTTTGCTTCCTGAACTCGC |
| ACO2-F | TTCTACATCCACGACGGCAG | CYSC1-F | ATCATCAGGAGCGAACACCG |
| ACO2-R | CGGATAGTGGCTGACCTTGG | CYSC1-R | TCGAACAGTGCAGACGACAA |
| ACS-F | CCAACATGGACGACGAGACA | DNMT1-F | AAAGAAGCGCAAAGCAAAGC |
| ACS-R | CTAGCGTGAACCATCGGTGA | DNMT1-R | GCACAACAGGCTCATCAGTG |
| PYRP2-F | GGCAGCACGCAAGGAGGTAA | metE-F | GGTGAGCAGCTCTCTGGTTT |
| PYRP2-R | GCAACCTCAAACATCTCACCATCA | metE-R | ACCTTGGCTGGTCATTCCTG |
| DHFR1-F | CTTCTCCCGCTGCTTCCTCG | TAT2-F | TCCAAGCTCTTGCTGACGAG |
| DHFR1-R | GCCTTGCCATCCAGCCTCA | TAT2-R | TAGGGTGAAGACAGGAGCCA |
| DHFR2-F | CGGTGGTAGAGAGCAACATTAGGC | CYSK-F | GGAGAAAGGGCTGATCTCGC |
| DHFR2-R | TAAGTTGAATCGCATCTGACACCC | CYSK-R | CAGGCATGACTGCAACGAAC |
| RFK-F | TCAAAGGATTTGGCCGTGGT |  |  |
| RFK-R | CACGTGTTGAAAGTCCAGCC |  |  |

**Table S3 The Raw data of mRNA-seq, data statistics and quality assessment after quality control**

| Sample | Raw Reads | Clean Reads | Error rate(%) | Q20(%) | Q30(%) | GC content(%) | Total mapped | Multiple mapped | Unique mapped |
| --- | --- | --- | --- | --- | --- | --- | --- | --- | --- |
| 0 mM | 119,721,474 | 118,664,398 | 0.0243 | 98.29 | 94.91 | 53.90 | 91.56 | 14.96 | 76.75 |
| 6 mM | 127,200,271 | 126,120,735 | 0.02415 | 98.36 | 95.07 | 53.09 | 89.14 | 12.74 | 76.40 |

**Table S4 The Raw data of miRNA-seq, data statistics and quality assessment after quality control**

| Sample | Raw Reads | Total reads | Clean Reads | Error rate(%) | Q20(%) | Q30(%) | GC content(%) | Useful reads(18-32nt) | Total mapped | Mapped reads(+) | Mapped reads(-) |
| --- | --- | --- | --- | --- | --- | --- | --- | --- | --- | --- | --- |
| 6 mM | 11,404,251 | 9,128,675 | 10,581,629 | 0.0231 | 98.76 | 96.1 | 51.29 | 9,128,675 | 5,655,202 | 3,402,122 | 3,007,966 |
| 0 mM | 10,620,690 | 9,064,921 | 10,238,530 | 0.023 | 98.82 | 96.26 | 48.3 | 9,064,921 | 5,721,117 | 3,431,400 | 3,057,490 |

**Table S5 The prediction of miRNA targeted mRNA related to folate metabolism pathway**

| miRNA | Target mRNA | Expectation | UPE$ | miRNA start | miRNA end | Target start | Target end | alignment | Inhibition |
| --- | --- | --- | --- | --- | --- | --- | --- | --- | --- |
| Nov-m0012-3p | MTRF(Si5g28250.1) | 2.5 | -1 | 1 | 24 | 570 | 593 | AUGGACUUGAGCUUUGCGGAUAUG  .:: :.::::::.:..:::.::: AGUACCUGCAAAGUUUGAGUUCAU | Cleavage |
| Nov-m0112-3p | PYRP2(Si7g20840.1) | 4 | -1 | 1 | 21 | 143 | 163 | CCUUGGCACGGGAUGCUGCCG  ::: :.:::.::::.: :: AUGCAACGUCCUGUGCUACGG | Cleavage |
| Nov-m0139-3p | DHFR1(Si2g40820.1) | 4 | -1 | 1 | 24 | 402 | 425 | AGUCGAGCUUGUUUAUGAAAUGCC  ::: ::.::::.:::..: CUCGCCUCAGAAGCAAGUUCGGUU | Cleavage |
| Nov-m0211-5p | MTRF(Si5g28250.1) | 4.5 | -1 | 1 | 24 | 422 | 445 | AUGUUACGUUCGGACGACCAUGCC  :: :::::::..:::.:: :.:  UGCCUGGUCGUUUGAAUGUCAUAC | Cleavage |
| Nov-m0211-5p | MTRF(Si5g28250.1) | 4.5 | -1 | 1 | 24 | 532 | 555 | AUAAUGAGUGGUAAUAAUGAGUGC  .::::::: .:.:::: :: AAAGAUAUUAUUAAUAUUCAUCAU | Translation |
| Nov-m0286-3p | 5-FCL2(Si7g29450.1) | 4.5 | -1 | 1 | 24 | 1167 | 1190 | UUUUCUGUGAAGCACUCGGCAAAG  :.:::::: :::: :.:.: UCACUCUGAGUGCCUCACUGGAGA | Translation |
| Nov-m0335-5p | MOCS1A(Si6g11970.1) | 4.5 | -1 | 1 | 24 | 458 | 481 | UUGCAGCUGUGGAAGAUAUGGUGU  .::::.::. . :::::::: CAGAUAUAUUUUUGGAAGCUGCAA | Translation |
| Nov-m0345-5p | RIBA2(Si3g21170.1) | 4.5 | -1 | 1 | 24 | 1296 | 1319 | AUAGUUUUGGUUUUGUCGAGUGUC  ... :.:::::.:.:::.: ::  UGUGAUUGACAAGAUCAAGAAUAA | Cleavage |
| Nov-m0604-3p | RIBA1(Si1g21120.1) | 5 | -1 | 1 | 21 | 896 | 916 | CCUUGGCACGGGAUGCUGCCG  :.::::: .::: ::::: UUUCGGCAUCAUGUGACAAGG | Translation |
| Nov-m0717-5p | FTHFD(Si1g22840.1) | 5 | -1 | 1 | 24 | 542 | 565 | AGUGGCCGCCGGCGCUGUAGCGGG :: :: ::::::: :::::: ::  CCGGCGACAGCGCGGGCGGCAACA | Translation |
| Nov-m0731-5p | DHFR2(Si8g12590.1) | 5 | -1 | 1 | 23 | 727 | 749 | UUUUCAGGGUUUGCCGAGUGUUU  : :: : .::::.:::.:: GUGAAGUCUGAGAACCUUGAGAA | Cleavage |
| miR171h-5p | MOCS1A(Si6g11970.1) | 5 | -1 | 1 | 21 | 295 | 315 | UGGUAUUGUUUCGGCUCAUGU  : :::.:::: .:: :::: GAAGGAGUCGAACUAACACCA | Cleavage |
| miR171k-5p | MOCS1A(Si6g11970.1) | 5 | -1 | 1 | 21 | 295 | 315 | UGGUAUUGUUUCGGCUCAUGU  : :::.:::: .:: :::: GAAGGAGUCGAACUAACACCA | Cleavage |
| miR319a-5p | RIBA2(Si3g21170.1) | 5 | -1 | 1 | 20 | 810 | 829 | GAGCUCUCUUCAGUCCACUC  .::: .::::: ::::.:  AGGUGUGCUGAAAAGAGUUG | Cleavage |
| miR319c-5p | RIBA2(Si3g21170.1) | 5 | -1 | 1 | 20 | 810 | 829 | GAGCUCUCUUCAGUCCACUC  .::: .::::: ::::.:  AGGUGUGCUGAAAAGAGUUG | Cleavage |

**Table S6 The prediction of miRNA targeted mRNA related to [methionine](javascript:;) metabolism pathway**

| miRNA | Target mRNA | Expectation | UPE$ | miRNA start | miRNA end | Target start | Target end | alignment | Inhibition |
| --- | --- | --- | --- | --- | --- | --- | --- | --- | --- |
| Nov-m0038-5p | ACS(Si7g21960.1) | 3.5 | -1 | 1 | 24 | 292 | 315 | AAUGAACUUGGCUAUUAUCUGAUU  ::. .::.:::::::::::  AGAGAGGCGAUGGCCAAGUUCAUG | Cleavage |
| Nov-m0064-5p | ADI1(Si9g24300.2) | 4 | -1 | 1 | 21 | 207 | 227 | CAUGCUUCAGCAACCUGGUGG  :: ::::: .::::::::: GCAGGAGGUUAUUGAAGCAUG | Translation |
| Nov-m0081-3p | metE(Si3g04370.1) | 5 | -1 | 1 | 24 | 677 | 700 | AAAUGCCGGAUUCAGGUGCGCCGC  : ::.:::.:::: : ::: ACACAGAACUUGAGUCCGCCCUUU | Cleavage |
| Nov-m0128-3p | APIP(Si8g12550.2) | 5 | -1 | 1 | 24 | 792 | 815 | UUGGGACUUUCGGAAUUCACAGCU  .: :::. :.:::: :::.:: CAUUUUGAGCCCUGAAAUUCCUAA | Cleavage |
| Nov-m0233-3p | metE(Si3g04370.1) | 5 | -1 | 1 | 24 | 888 | 911 | CUAAGAUCGACAGACCAGGAGCGU  : .:::: ::: .::::::.  CAACAUCUGGGCUGAUGAUCUUGC | Translation |
| Nov-m0244-5p | metE(Si3g04370.1) | 4.5 | -1 | 1 | 20 | 1348 | 1367 | UUCGCUCUGUGCUCACCACG :: :::::.::: .::::: CGCCGUGAGUACAAGGCGAA | Cleavage |
| Nov-m0286-3p | TAT2(Si8g15570.1) | 5 | -1 | 1 | 24 | 519 | 542 | UUUUUCUCGGUUAGGUGCUUGGCC  ..: : .::: :.::::::.: CUAUGAUCUUCUACCUGAGAAAGA | Translation |
| Nov-m0319-3p | SRM(Si1g02540.2) | 5 | -1 | 1 | 24 | 1037 | 1060 | ACUUCGCUUCAGGAUUGGGAUGGU  .: ::: :.::.::..: ::.:: UUCUUCCAAGUCUUGGGGAGAGGU | Cleavage |
| Nov-m0330-3p | APIP(Si8g12550.2) | 5 | -1 | 1 | 21 | 7 | 27 | AUCGUGGACGCACCAGAUGCA :::. : :::::: :..:::: UGCGGCGGGUGCGCCUGCGAU | Cleavage |
| Nov-m0372-5p | AK(Si6g12370.2) | 5 | -1 | 1 | 24 | 349 | 372 | UCCUUGGCGGACAACCAGAUCUAG  :::.: :::::. :::: ::: UCUGAUUUUGUUGUUGGCCAUGGA | Cleavage |
| Nov-m0389-3p | BHMT2(Si9g47530.1) | 5 | -1 | 1 | 24 | 315 | 338 | CUCGAUUGCCUCACUAUAGGCAGC  :.::: : ::::: :.::::  GAAGUCUACACUGAGGAAGUCGAA | Cleavage |
| Nov-m0461-5p | CYSC1(Si7g02940.1) | 5 | -1 | 1 | 24 | 264 | 287 | UUGAAGGCUGGUUCCACGAGGUAC  : .:: :: :.::. ::::::. CGAGUUCCUGCAGCCGUCCUUCAG | Cleavage |
| Nov-m0470-3p | APIP(Si8g12550.2) | 4.5 | -1 | 1 | 24 | 99 | 123 | ACCGUUCGAUGGCU-GAUAAGUCCC  .:::.:: ::::: :::.:::: CACGCUUGUCGAGCCAACGAGCGGU | Cleavage |
|  | CYSK(Si4g07980.1) | 5 | -1 | 1 | 24 | 402 | 425 | ACCGUUCGAUGGCUGAUAAGUCCC  :: ::::::: :::.::  UGUCAUUCACAGCCAUGGAAUGGA | Cleavage |
| Nov-m0481-5p | ACS(Si7g21960.1) | 4.5 | -1 | 1 | 20 | 774 | 793 | AGCUGCACCUGGGGAUGCCC  : :.:::::.:::::: :  CGACGUCCCCGGGUGCAACC | Cleavage |
| Nov-m0492-5p | DNMT1(Si9g05970.1) | 4.5 | -1 | 1 | 23 | 3374 | 3396 | UUCGCCGAGUGUUUUAUUAAAUA  :.. .::.::.:.::.::: CUGCUGGGGAAGCAUUUGGUGAA | Cleavage |
| Nov-m0664-3p | APIP(Si8g12550.2) | 5 | -1 | 1 | 24 | 266 | 289 | UUGCAGCUGUGGAAGAUAUGGUGU  :::.: : .::..::::::. AGGACAUGUAUGUCAUGGCUGCAG | Cleavage |
| Nov-m0677-5p | ACS(Si7g21960.1) | 5 | -1 | 1 | 24 | 1284 | 1307 | AUUUGCAGCUGUGUGAAGUGAGUG  .::::...:::: :::.:  CCGCAGCUUCGUGCAGCAGCAGAA | Cleavage |
|  | DNMT1(Si9g05970.1) | 5 | -1 | 1 | 24 | 2356 | 2379 | AUUUGCAGCUGUGUGAAGUGAGUG  ::: ::: ::::::::: ACUGUUCUUGACAAUGCUGCAAAU | Translation |
| Nov-m0717-5p | CYSK(Si4g07980.1) | 4 | -1 | 1 | 23 | 340 | 362 | UUUUCAGGGUUUGCCGAGUGUUU  : :: : ::.:::::::::.  GCAAACGCAGCGAACCCUGAAGC | Cleavage |
| Nov-m0784-3p | ACS(Si7g21960.1) | 5 | -1 | 1 | 21 | 972 | 992 | CUGAGGAGCGAGGCCAUGAUG : :::::::: :.:::::.  CUUCAUGGCCCGGUUCCUCGC | Translation |
| Nov-m0797-3p | metE(Si3g04370.1) | 4.5 | -1 | 1 | 23 | 2207 | 2229 | GAUCUCCUUGUCCUUGCCGGUGG  ::: ::::: ::: ::::.:: AGACCCGCAAGUACACGGAGGUC | Cleavage |
| miR528a-5p | AMD1(Si7g16790.2) | 3.5 | -1 | 1 | 21 | 119 | 139 | UGGAAGGGGCAUGCAGAGGAG  :.:::.:::::.::::: GUGGUUUGCGUGCCCUUUCCA | Cleavage |
| miR528b-5p | AMD1(Si7g16790.2) | 3.5 | -1 | 1 | 21 | 119 | 139 | UGGAAGGGGCAUGCAGAGGAG  :.:::.:::::.::::: GUGGUUUGCGUGCCCUUUCCA | Cleavage |
